# Supplementary material for: ActSeek: fast and accurate search algorithm of active sites in alphafold database
Source: Bioinformatics. 2025 Jul 26;41(8):btaf424. doi: 10.1093/bioinformatics/btaf424 (PMC12343037; doi:10.1093/bioinformatics/btaf424)
Supplement: btaf424_Supplementary_Data [file btaf424_supplementary_data.pdf]

Erlotinib

	ActSeek	Structmotif-search	pyScoMotif	ProBis	FoldDisco
ActSeek	190				
Structmotif-search	58	3935			
Pyscomotif	21	24	132		
ProBis	56	82	12	249	
Folddisco	33	33	20	21	194

Sorafenib

	ActSeek	Structmotif-search	pyScoMotif	ProBis	FoldDisco
ActSeek	14				
Structmotif-search	8	2479			
Pyscomotif	10	156	2045		
ProBis	6	30	21	305	
Folddisco	8	25	44	9	233

Beta blockers

	ActSeek	Structmotif-search	pyScoMotif	ProBis	FoldDisco
ActSeek	29				
Structmotif-search	4	10			
Pyscomotif	6	3	6		
ProBis	4	1	1	13	
Folddisco	18	5	6	5	1737

Table 1: The three tables showing the number of results each program found in each case and their intersections.
